# Molecular Dynamics Simulation of Membrane Distillation for Different Salt Solutions in Nanopores

**DOI:** 10.3390/molecules29194581

**Published:** 2024-09-26

**Authors:** Jiadong Li, Yuanhe Ding, Jinyi Qin, Chuanyong Zhu, Liang Gong

**Affiliations:** College of New Energy, China University of Petroleum (East China), Qingdao 266580, China; jd.li@upc.edu.cn (J.L.); upcdyh@163.com (Y.D.); z22150084@s.upc.edu.cn (J.Q.)

**Keywords:** nanoporous membrane, salt solution, direct contact membrane distillation, non-equilibrium molecular dynamics

## Abstract

Nanoporous membranes offer significant advantages in direct contact membrane distillation applications due to their high flux and strong resistance to wetting. This study employs molecular dynamics simulations to explore the performance of membrane distillation in a single nanopore, mainly focusing on wetting behavior, liquid entry pressure, and membrane flux variations across different concentrations and types of salt solutions. The findings indicate that increasing the NaCl concentration enhances the wetting of membrane pores, thereby decreasing the entry pressure of the solution. However, at the same salt concentration, the differences in wetting and liquid entry pressure among various salts, including CaCl_2_, KCl, NaCl, and LiCl, are minimal. The presence of hydrated ions significantly reduces membrane flux. As the concentration of NaCl solutions increases, the number of hydrated ions rises, thereby lowering the membrane flux of the salt solution. Furthermore, the type of salt has a pronounced effect on the structure of hydrated ions. Solutions with Ca^2+^ and Li^+^ exhibit the smallest first-layer radius of hydrated ions. Under the same salt concentration, KCl solutions demonstrate the highest membrane distillation flux, while CaCl_2_ solutions show the lowest flux.

## 1. Introduction

Desalination serves as a critical method for addressing global water scarcity and can be classified into membrane-based and non-membrane-based processes. Compared to traditional non-membrane-based technologies such as distillation and flash evaporation, membrane-based approaches offer notable advantages regarding energy consumption and capital costs [1,2,3]. Among membrane desalination technologies, reverse osmosis and membrane distillation are widely recognized. Reverse osmosis operates by using pressure to propel water molecules through a semipermeable membrane, necessitating high pressure to overcome osmotic pressure and resulting in substantial pump energy consumption. Conversely, membrane distillation utilizes hydrophobic porous membranes to achieve the desalination process. The underlying principle is that only water vapor can permeate through the micropores under normal pressure. In the presence of a temperature gradient across the membrane, vapor pressure compels water vapor to migrate toward the lower temperature side, where it condenses. This technology can harness low-grade thermal energy from industrial waste heat, thereby mitigating the high energy consumption associated with the desalination industry [4,5,6].

Traditional polymer microporous membranes, while widely used in large-scale industrial applications [7], exhibit vulnerabilities to wetting and suffer from a rapid decline in membrane flux, presenting substantial operational challenges. With the evolution of nanotechnology, nanoporous materials, particularly carbon nanotube (CNT) membranes, have emerged as a focal point of research interest due to their superior water flux and enhanced resistance to wetting [8,9]. Notably, CNT-based membranes have demonstrated significant benefits in direct contact membrane distillation applications, owing to their high water flux and robust wetting resistance [10]. This interest has spurred several studies leveraging experiments and molecular dynamics simulations to scrutinize the performance of nanoporous membranes in membrane distillation processes. For instance, An et al. [11] demonstrated the potential of membrane distillation as an emerging desalination technology by developing membranes with ideal material properties. They anchored functionalized CNTs to nanofibers of electrospun membranes. Covalent modification and fluorination of CNTs improved their dispersibility and interfacial interaction with the polymer membrane, resulting in well-aligned CNTs inside crystalline fibers with superhydrophobic properties. Tijing et al. [12] fabricated a kind of superhydrophobic CNT-loaded membrane via one-step electrospinning, and the results from direct contact membrane distillation showed that the CNT-loaded membrane exhibited greater liquid entry pressure and higher membrane flux compared to the nanofiber membranes without CNTs. Gethard et al. [13] synthesized membranes using CNTs and polyvinylidene fluoride (PVDF) as the matrix. They found that immobilizing CNTs within the pores of a hydrophobic membrane positively modifies water–membrane interactions, enhancing vapor permeability while preventing liquid from penetrating the membrane pores. The incorporation of nanotubes resulted in a 1.85 times increase in flux and 15 times improvement in salt reductions. Bhadra et al. [14] developed a graphene oxide immobilized membrane for membrane distillation, demonstrating significant enhancements in flux and mass transfer coefficients. The above experimental studies have demonstrated that nanoporous membranes possess excellent water flux and salt rejection rates, showing great potential for development in membrane distillation applications. Since the process involves mass transfer at the nanoscale, the simulation work primarily relies on molecular dynamics simulations. Wei et al. [15] investigated water transport through graphene oxide membranes using molecular simulations and theoretical analysis. They considered flow through interlayer galleries, expanded channels like wrinkles, and pores within the sheet. Their findings revealed that while nanoconfinement can enhance flow, fast water transport through pristine graphene channels is hindered by a side-pinning effect from capillaries in oxidized regions. Kieu et al. [16] focused on the evaporation behavior of water through the capillary channel of a vertically aligned graphene bilayer using molecular dynamics simulations. By tuning the surface wettability via surface charge states, they examined structural and environmental parameters, such as capillary channel distance and temperature. The results indicated significant evaporation enhancement when the graphene bilayer contacts the water surface. A pioneering study by Zhang et al. [17] explored the mass transfer mechanisms in direct contact membrane distillation through double-layer graphene channels using molecular dynamics simulations, revealing that the permeation flux is nearly three orders of magnitude higher than that of currently commonly used microporous polymer membranes. Norouzi and Park [18] conducted studies on CNT membranes, revealing the exceptional potential of nanopores as efficient channels for membrane distillation. Their findings indicate that the simulated water vapor diffusion coefficient exceeds the results of the Knudsen diffusion model by approximately an order of magnitude, with membrane flux being two orders of magnitude higher than that of conventional microporous polymer membranes. Zhu et al. [19] utilized molecular dynamics simulations to investigate the water distillation process within individual nanopores. The research findings indicate that the permeation flux through nanopores is three orders of magnitude greater than that through microporous membranes. These investigations have markedly advanced the comprehension of the intrinsic mechanics governing nanopore-based membrane distillation. However, there remains a gap in the research, as these studies have not yet broadened their scope to encompass distillation processes involving various salt solutions.

Therefore, this paper will employ non-equilibrium molecular dynamics simulations to investigate the membrane distillation process in single nanopores, emphasizing the influence of salt concentration and salt type on the distillation efficiency and wettability. The structure of the paper is organized as follows: Section 2 introduces the physical model and outlines the simulation methodologies employed. Section 3 delves into a detailed analysis of how salt concentration and type affect wettability, liquid entry pressure, the structure of hydrated ions, and ultimately membrane flux. The concluding section will synthesize the findings of this study, presenting the implications and contributions to the field of membrane distillation.

## 2. Results and Discussion

### 2.1. Contact Angle of Salt Solution Droplets and LEP

**Figure 1:**
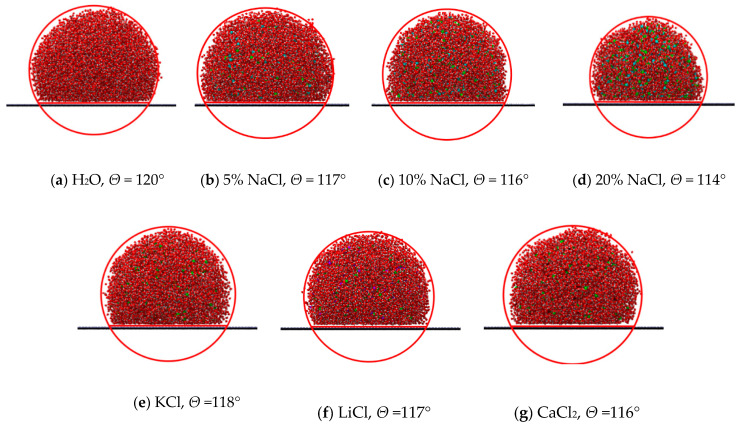
Molecular snapshot of contact angle of droplet.

**Figure 2:**
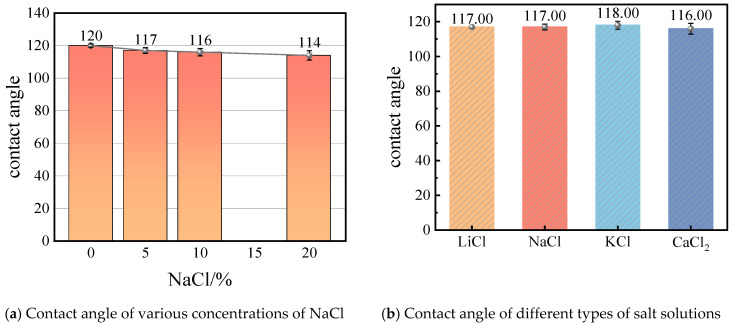
Contact angle in different concentrations of NaCl and various salt solutions.

Figure 1a–d illustrate the contact angles of various concentrations of NaCl solutions on the wall surfaces. As observed in this figure, with the increase in salt concentration, there is a decrease in the contact angle of the droplets. This phenomenon indicates that the wetting properties of salt solutions strengthen with higher salt concentrations. Furthermore, Figure 1e–g present the contact angles of different types of salt solutions at a concentration of 5% on graphene surfaces. The impact of various salts on the contact angle appears to be relatively minimal. Among them, the solution of KCl shows the highest contact angle, reaching up to 118°. Conversely, the CaCl_2_ solution exhibits the smallest contact angle, approximately 116°. The contact angles of NaCl and LiCl solutions are intermediate. It is noted that although the differences in contact angles are minimal, between 1–3 degrees, even slight variations can significantly impact the wettability of the membrane surface. This affects the LEP and the potential for pore wetting, which are critical for maintaining membrane performance in high-salinity environments. Thus, these small differences in contact angles can have important practical implications. Figure 2 shows the contact angles for different concentrations of NaCl and various salt solutions. To address the potential impact of line tension on the contact angle, we have minimized this effect by measuring droplets of different sizes and included error bars in the results. Additionally, using cylindrical droplets is another method that can be employed to reduce the influence of line tension on the contact angle [20]. In this study, error bars were calculated based on data from multiple repeated simulations. Specifically, for each dataset, we conducted n independent simulations/experiments and calculated the mean and standard deviation. The error bars represent the standard error of the mean (SEM). It is noted that although the differences in contact angles are minimal, between 1–3 degrees, even slight variations can significantly impact the wettability of the membrane surface. This affects the LEP and the potential for pore wetting, which are critical for maintaining membrane performance in high-salinity environments. Thus, these small differences in contact angles can have important practical implications.

**Figure 3:**
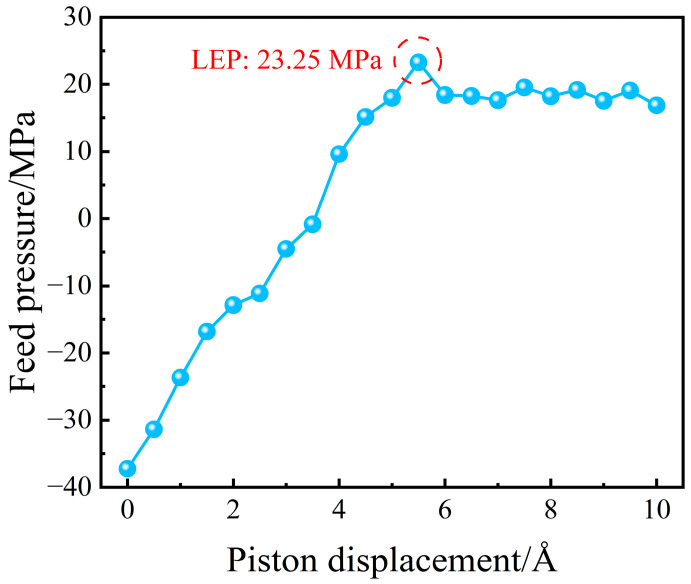
The relationship between the feed pressure and the piston displacement.

Figure 3 depicts the relationship between the LEP of pure water and the displacement of the piston. Initially, the water molecules in the liquid phase are relatively distant from both the piston and the membrane surface, resulting in a tensile force for water from the upper piston and the membrane surface. Consequently, the piston experiences a negative force from the water molecules, creating a negative pressure zone. As the piston’s displacement gradually increases, the interaction force between the piston and the liquid phase shifts from attraction to repulsion, causing the pressure to become positive progressively and continually rise. At a piston displacement of 5.5 Å, the pressure of the liquid phase reaches its maximum value of 23.25 MPa. This observation suggests that when the piston displacement exceeds 5.5 Å, the liquid begins to enter the pore spaces, indicating that the LEP under these conditions is 23.25 MPa. This finding is critical for understanding the threshold at which nanoporous membranes begin to allow liquid penetration.

**Figure 4:**
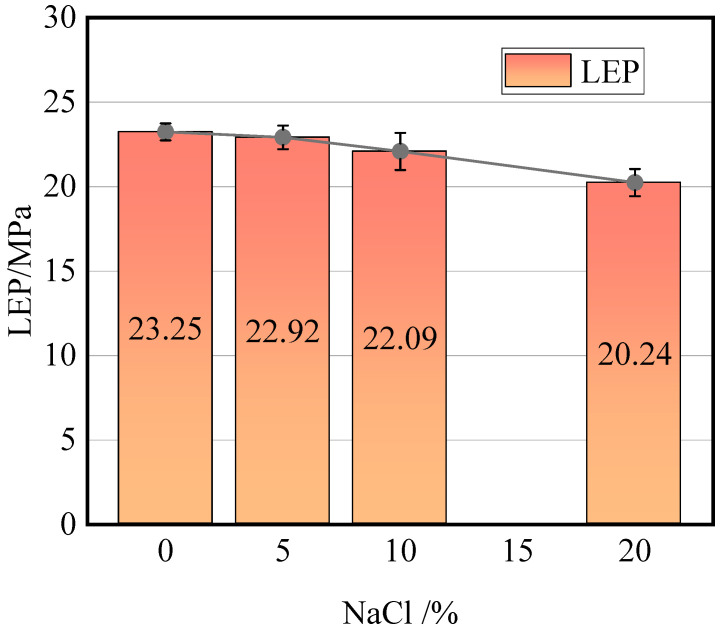
Effect of NaCl mass fraction of feed on LEP.

Figure 4 illustrates the relationship between the LEP of the nanopore and the salt concentration in the feed solution. As the salt concentration in the feed solution increases, the LEP of the nanopore gradually decreases, with the rate of decrease progressively accelerating. This signifies that higher salt solution concentrations lead to a more pronounced wetting of the membrane pores. This is because the enhancement in wettability significantly lowers the energy barrier for water molecules, allowing them to permeate more readily into the nanoporous structure. Specifically, as the mass fraction of NaCl in the feed solution rises from 0% to 20%, the LEP of the nanopore decreases by 3.01 MPa, corresponding to a reduction of 12.9%.

**Figure 5:**
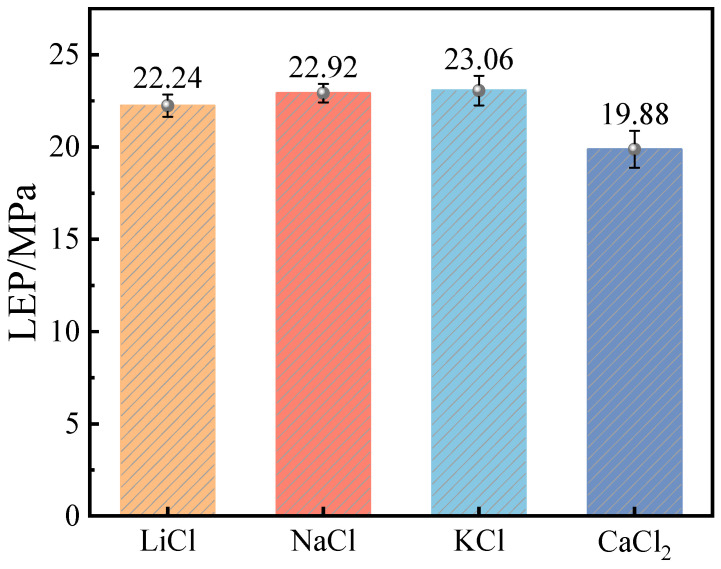
LEP of different kinds of saline water.

Figure 5 demonstrates the LEP for various salt solutions at a concentration of 5%. The salts examined include CaCl_2_, LiCl, NaCl, and KCl. The LEP values for these salt solutions are arranged in ascending order as CaCl_2_ < LiCl < NaCl < KCl. This ordering again signifies that stronger wetting properties correspond to a lower LEP. Compared to pure water, which has an LEP of 23.25 MPa under the same conditions, the LEP values for CaCl_2_, LiCl, NaCl, and KCl show respective decreases of 14.5%, 4.3%, 1.4%, and 0.8%. Notably, the most significant decrease is observed with CaCl_2_, and it can be anticipated that adding more CaCl_2_ would result in a further decrease in LEP. This is attributed not only to the pronounced effect of Ca^2+^ ions on the wetting properties of the solution but also to the fact that the molar concentration of Cl^−^ ions in CaCl_2_ is twice that of other salts with the same molar fraction. Consequently, this leads to the most substantial reduction in LEP.

### 2.2. The Structure of Hydrated Ions

**Figure 6:**
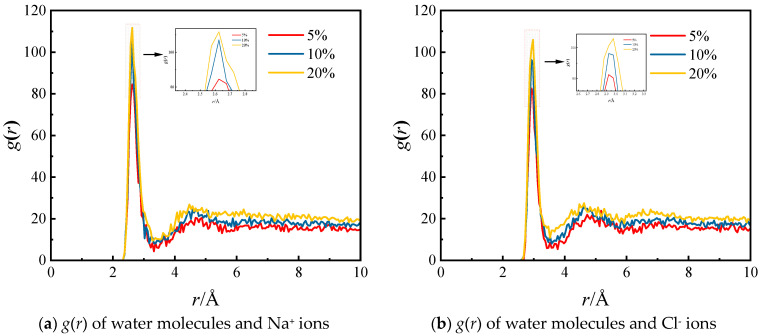
RDFs of water molecules and salt ions of salt solutions with different NaCl mass fractions.

Due to the formation of hydrated ions, salt ions bind with surrounding water molecules, thereby affecting the evaporation capacity of the solution and consequently influencing membrane flux. Therefore, this section investigates the structure of hydrated ions of different salt ions. Salt ions in water form hydrated ions by binding to surrounding water molecules, comprising the central ion’s first and second hydration shells. The structural arrangement of hydrated ions can be quantitatively demonstrated using radial distribution functions (RDFs). Figure 6 illustrates the RDFs of water molecules and NaCl solutions with varying mass fractions of NaCl. In Figure 6a, The first peak in the graph occurs at *r* = 2.625 Å, with peak values of 84.5, 107.1, and 111.7 for NaCl solutions with mass fractions of 5%, 10%, and 20%, respectively. Meanwhile, the second peak, located near *r* = 4.6 Å, exhibits peak values of 20.4, 24.5, and 25.0 for the corresponding concentrations. The first peak represents the first hydration shell of the hydrated ions, while the second peak and the subsequent region signify the second hydration shell, with the valley between the two peaks corresponding to the radius of the first hydration layer. With an increase in salt concentration, the peak values also rise, indicating a higher quantity of water molecules within the structure of Na^+^ hydrated ions and stronger interactions between water molecules and Na^+^. Figure 6b displays the RDF *g*(*r*) of water molecules and Cl^−^, revealing a main peak and two minor peaks. The primary peak, located near *r* = 2.95 Å, exhibits peak values of 82.6, 96.3, and 105.9 for solutions of different concentrations. The second and third minor peaks are situated near *r* = 4.7 Å and *r* = 6.8 Å, respectively. Similarly, with an increase in salt concentration, the peak values rise, indicating a higher quantity of water molecules within the structure of Cl^−^ hydrated ions and stronger interactions between water molecules and Cl^−^. Comparing the peak values of Na^+^ and Cl^−^, it is evident that the first peak of Na^+^ is more advanced and possesses a higher peak value. This observation suggests that the first hydration shell of Na^+^ has a greater density, and stronger interaction strength, and the surrounding water molecules are less likely to evaporate.

**Figure 7:**
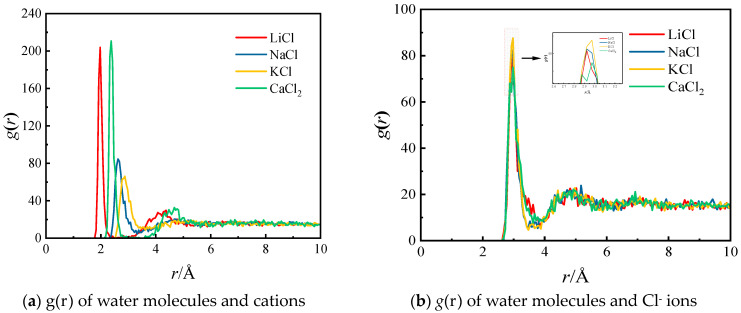
RDFs of water molecules and salt ions of salt solutions with different kinds of salts.

Figure 7a elucidates the significant differences in the *g*(*r*) between water molecules and various cations. The radii at which the first peak occurs for each cation are Li^+^ (*r* = 1.975 Å), Ca^2+^ (*r* = 2.375 Å), Na^+^ (*r* = 2.625 Å), and K^+^ (*r* = 2.875 Å), with peak values of 203.8, 210.7, 84.5, and 66.1, respectively. The radii of the first hydration layers for Li^+^, Na^+^, K^+^, Ca^2+^, and Cl^−^ ions were determined to be 2.85 Å, 3.3 Å, 3.6 Å, 3.15 Å, and 3.7 Å, respectively. Notably, the hydration ion of Li^+^ exhibits the smallest radius for its first hydration layer, while the Ca^2+^ ion’s first hydration layer contains the highest number of water molecules. This suggests that both Li^+^ and Ca^2+^ ions demonstrate considerable strength in their hydration ion interactions. The significant differences in the hydration structures of these cations provide valuable insights into the varying behaviors of salt solutions at the molecular level. Figure 7b depicts the *g*(*r*) of water molecules and Cl^−^ in different types of salt solutions. The first peak still appears near *r* = 2.95 Å but with varying peak values. The first peak values for LiCl, NaCl, KCl, and CaCl_2_ are 81.7, 82.6, 87.6, and 74.9, respectively. This indicates that while different cations do not affect the radius of Cl^−^’s first hydration layer, they do influence the strength of hydration ion interactions by affecting the number of water molecules within the first hydration layer. It is worth noting that although CaCl_2_ has the smallest first peak value, the quantity of Cl^−^ ions in the solution is twice that of the other three salt solutions; thus, Ca^2+^ still has a significant influence on the strength of Cl^−^’s hydration ion interaction.

### 2.3. Membrane Flux

**Figure 8:**
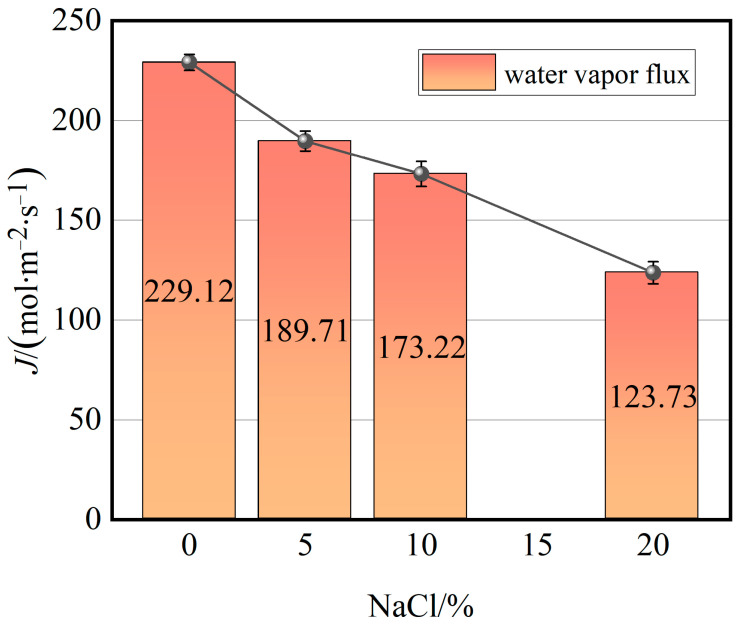
Effect of NaCl mass fraction of feed on water vapor flux.

As the water molecules evaporate and pass through the nanopores, the membrane flux is influenced by the concentration and type of salt solutions. Figure 8 illustrates the correlation between the membrane flux and NaCl mass fraction. As the NaCl mass fraction increases, the membrane flux demonstrates an almost linear decrease. The membrane flux is predominantly influenced by the evaporation rate of water molecules in the feed solution and the diffusion rate of water vapor molecules in the membrane pores. In the direct contact membrane distillation process, salt ions do not enter the membrane pores, thereby not affecting the diffusion rate of water vapor molecules within the membrane pores. Consequently, the evaporation rate of water molecules in the feed solution primarily influences the membrane flux. As previously discussed, with the increase in NaCl concentration, the number of hydrated ions increases, making the evaporation of water more challenging [21]. This phenomenon can be attributed to the increased hydration, which effectively reduces the free water molecules available for evaporation. The hydrated ions create a ‘binding’ effect, where water molecules are more likely to remain in the liquid phase rather than transition into vapor.

**Figure 9:**
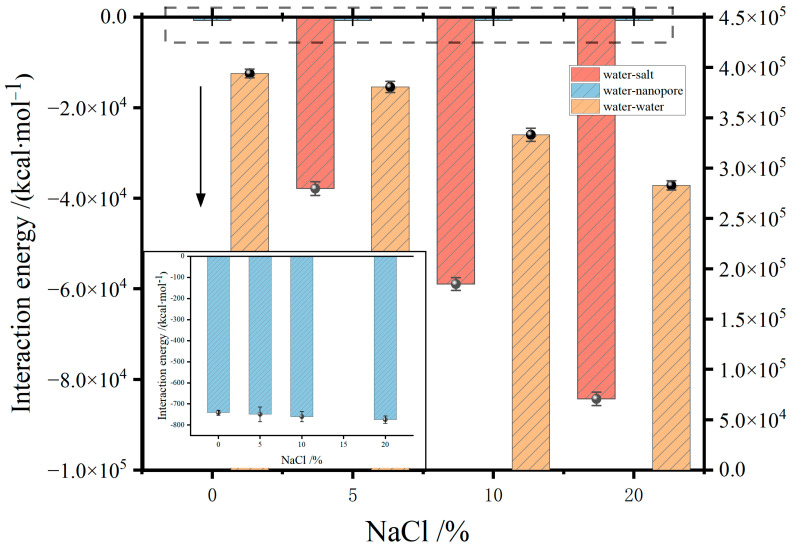
Effect of NaCl mass fraction of feed on the interaction energy.

Figure 9 illustrates the interaction energies between water molecules and molecules of membrane pores, salt, and water in the feed solution, respectively. The interaction energies for water–nanopore and water–salt are negative, indicating attractive forces, while the water–water interaction energy is positive, indicating repulsive forces between water molecules. As the mass fraction of NaCl increases, both the water–membrane pore and water–salt interactions strengthen, whereas water–water interactions weaken. The enhanced water–membrane pore interaction promotes water molecule evaporation. Conversely, the strengthened water–salt interactions and weakened water–water interactions reduce the evaporation rate. When the NaCl mass fraction increases from 0 to 20%, the water–nanopore interaction energy changes by only −33.06 kcal·mol^−1^. In contrast, the water–water interaction energy decreases by 111,244.18 kcal·mol^−1^, and the water–salt interaction energy decreases by 46,478.75 kcal·mol^−1^ from 5% to 20%. This indicates that the ion–dipole attraction between NaCl and water molecules is stronger than the hydrogen bonding among water molecules. Consequently, more energy is required to break the ion–dipole attractions, thereby reducing the evaporation rate of water molecules at the convex liquid surface.

**Figure 10:**
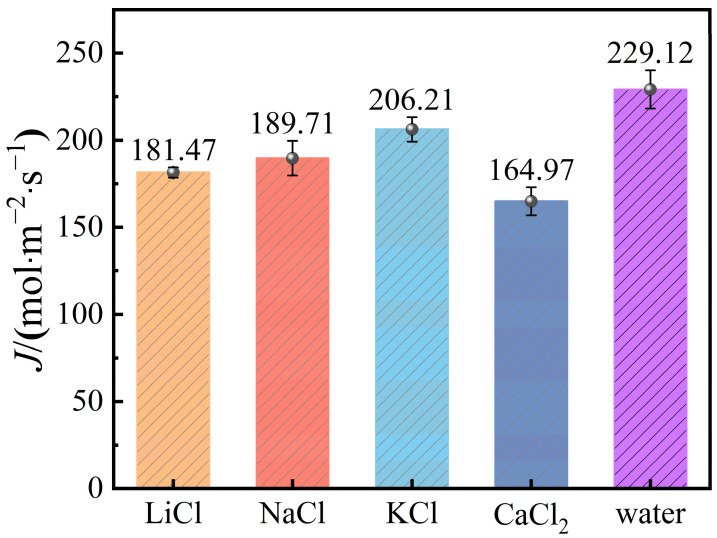
Water vapor flux of different kinds of saline water.

Figure 10 illustrates the membrane flux of different salt solutions at a salt concentration of 5%. The data reveal a clear trend in membrane flux across the four types of salt solutions, arranged in ascending order: CaCl_2_ < LiCl < NaCl < KCl. Compared to pure water, the membrane flux decreases by 28%, 20.8%, 17.2%, and 10% respectively for these solutions. The most significant decrease in membrane flux is observed in the CaCl_2_ solution. This can be attributed to the strong interaction between Ca^2+^ ions and water molecules. Ca_2+_ ions have a high charge density, leading to the formation of very stable and tightly bound hydration shells with the highest number of water molecules in the first hydration layer (see Figure 10), indicating considerable strength in their hydration ion interactions. Additionally, at the same molar concentration, CaCl_2_ contributes more Cl^−^ ions compared to other salts, resulting in a higher ion concentration in the feed solution. This increased ion concentration makes the evaporation of water molecules more difficult.

**Figure 11:**
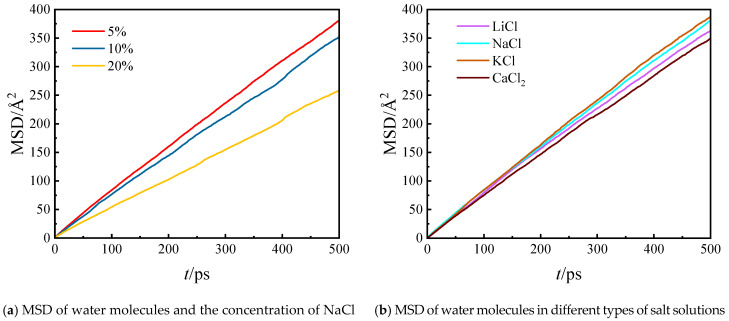
The MSD of water molecules and salt ions of different kinds of saline water.

**Figure 12:**
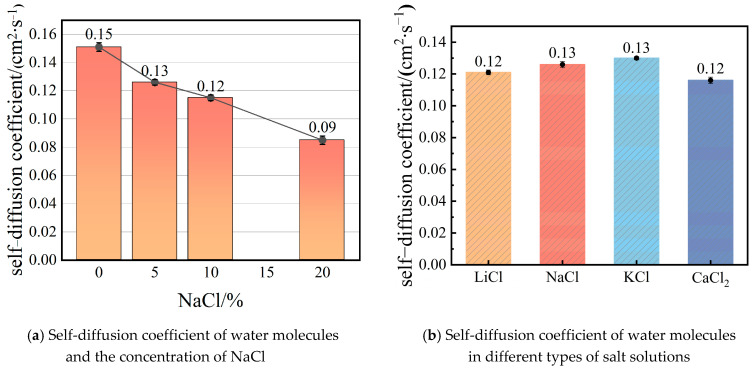
The self-diffusion coefficient of water molecules and salt ions of different kinds of saline water.

The evaporation capacity of liquid water is closely related to the diffusivity of water molecules. Higher diffusivity of water molecules facilitates easier evaporation [12]. Figure 11 presents the mean square displacement (MSD) of water molecules in different salt solutions, indicating their diffusive abilities. Figure 11a depicts the relationship between the MSD of water molecules and the concentration of NaCl. It shows that as the salt concentration increases, the diffusivity of water molecules decreases. The self-diffusion coefficients of water molecules at different NaCl concentrations are also reported in Figure 12a. It is noted that the self-coefficient of water molecules is 0.15 cm^2^·s^−1^ when the NaCl concentration is 0. Norouzi and Park [18] found that the self-diffusion coefficient at an average system temperature of 323K is approximately 0.17 cm^2^·s^−1^, and it increases as the temperature rises. Since the system temperature in our system is around 300K, the self-diffusion coefficient is slightly lower in this case. The decline in diffusivity in Figure 12a can be attributed to the increased interaction between water molecules and ions. As the NaCl concentration rises, more Na^+^ and Cl^−^ ions are present in the solution. These ions attract the surrounding water molecules, forming stable hydration shells through ion–dipole interactions. These interactions significantly restrict the movement of the water molecules, effectively reducing their mobility. Consequently, the diffusivity of water molecules decreases, which is reflected in the reduced MSD values. This phenomenon is also evident in the interaction energies shown in Figure 12, where increased ion concentrations lead to stronger water–ion interactions and weaker water–water interactions. These combined effects contribute to the observed reduction in water molecule mobility at higher salt concentrations. Figure 11b demonstrates the MSD of water molecules in different types of salt solutions. The order of diffusivity strength in these solutions is as follows: CaCl_2_ < LiCl < NaCl < KCl, which is consistent with the self-diffusivity coefficients presented in Figure 12b. This trend further explains why the membrane flux decreases with increasing NaCl concentration and why KCl and CaCl_2_ solutions exhibit the highest and lowest membrane flux, respectively.

## 3. Numerical Details and Theory

### 3.1. Water and Salt Molecular Model Validation

This study utilized LAMMPS software for conducting molecular dynamics simulations and employed OVITO software for visualization. The investigation focused on various salt solutions, specifically NaCl, KCl, CaCl_2_, and LiCl, to explore their effects on membrane distillation processes. To simulate water molecules, the study adopted the extended simple point charge model (SPC/E) [22], which is renowned for its accurate representation of water’s macroscopic properties at the gas–liquid interface. This model integrates the Lennard–Jones potential to account for interactions between oxygen atoms, and it includes electrostatic forces resulting from the charges on hydrogen and oxygen atoms [22]. These features enable the SPC/E model to accurately depict the surface tension [23] and vapor pressure of water [24], as well as to effectively capture the evaporation dynamics of both pure water [25,26,27] and salt solutions [28]. The parameters specified for the SPC/E model are a charge of +0.4238e for hydrogen atoms, −0.8476e for oxygen atoms, a hydrogen-oxygen bond length of 1.0 Å, and a bond angle of 109.47° [29]. The interaction potential for water molecules within this model is delineated as follows [22,29]:

(1) $$U(r_{ij})=4\varepsilon_{ij}[{\left(\frac{\sigma_{ij}}{r_{ij}}\right)}^{12}-{\left(\frac{\sigma_{ij}}{r_{ij}}\right)}^{6}]+\frac{q_{i}q_{j}}{4\pi \varepsilon_{0}r_{ij}}$$

where the L-J parameters for oxygen atoms are set as *ε_O-O_* = 0.1553 kcal·mol^−1^ and *σ_O-O_* = 3.166 Å, respectively. *q_i_* and *q_j_* are the atomic charges of the atoms *i* and *j*, and *ε*_0_ = 8.85 × 10^−12^ F/m represents the vacuum permittivity.

The interactions in water molecules included van der Waals’ forces and Coulomb’s forces. The interactions between carbon nanotubes, C atoms, and the piston with water molecules only involved van der Waals forces. The interaction parameters for the simulation are listed in Table 1. All other Lennard–Jones (L-J) parameters also followed the Lorentz–Berthelot rule [30]. The compositions of the four concentrations of NaCl solution are listed in Table 2. The LiCl, KCl, and CaCl_2_ solutions adopt the same salt molecule numbers as the NaCl solution.

### 3.2. The Physical Model for Direct Contact Membrane Distillation (DCMD)

**Figure 13:**
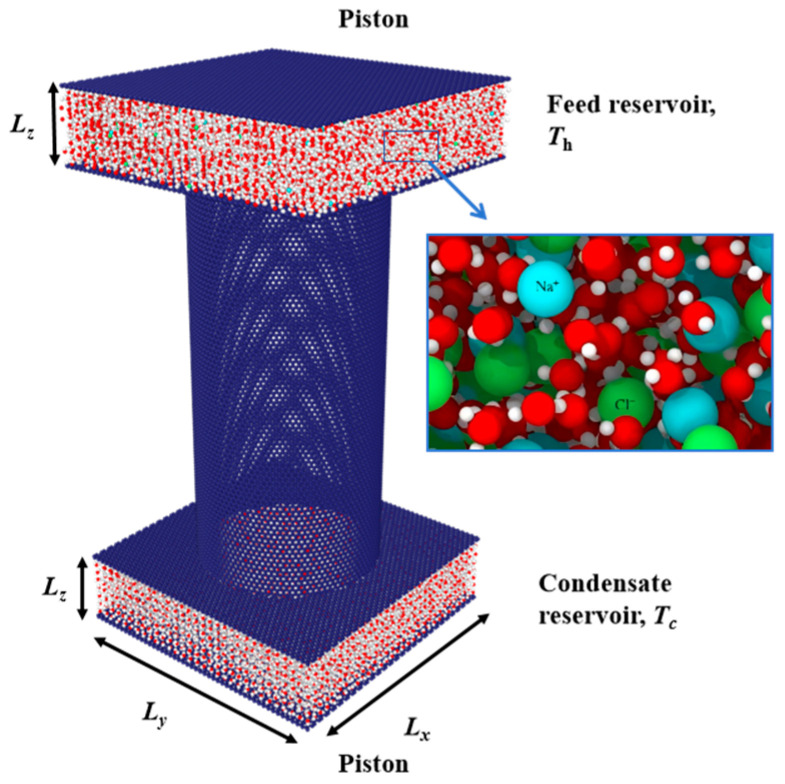
The physical model of the DCMD system for NaCl solution.

The DCMD system consists of a single nanopore and two reservoirs, as shown in Figure 13. In this model, the nanopore is composed of a carbon nanotube with a dimension of *L* = 17.04 nm and *d* = 7.16 nm. The upper reservoir is the hot water reservoir corresponding to the feed liquid, and the lower reservoir is the cold water reservoir corresponding to the permeation liquid. Between the CNT and the reservoirs is a square graphene sheet serving as the membrane surface. This graphene sheet has a circular region of carbon atoms removed and is assembled with the CNT to form a complete single-pore CNT membrane. At the top and bottom of the model are two complete square graphene sheets, which function as pistons to control the pressure of the hot and cold reservoirs. The CNT membrane pores and the carbon atoms on the graphene membrane surface remain stationary, with no consideration given to the temperature of the solid walls or their thermal motion affecting the transport of water molecules across the membrane. The dimensions of these reservoirs are *L_x_* × *L_y_* × *L_z_* = 120 Å × 120 Å × 30 Å.

In this model, the atoms in the feed and permeation reservoirs are controlled using Langevin thermostats to maintain temperatures of 320 K and 280 K, respectively. Meanwhile, the system as a whole is simulated within the NVE (microcanonical) ensemble, which conserves the total energy of the entire system. This approach allows for localized temperature control in specific regions while adhering to the overall energy conservation of the system. At the beginning of the MD simulation, the water molecules and salt molecules inside the feed reservoirs were equilibrated for 1 ns to reach a preset temperature. The non-equilibrium molecular dynamics simulations have a time step of 1 fs, lasting for a total of 5 ns, with the increment of water molecules on the condensation side being recorded every 0.5 ns. The permeate flux can be calculated using the following equation [17]:

(2) $$J_{MD}=\frac{\Delta N}{S\cdot \Delta t}$$

where *J*_MD_ is the permeate flux of water mol/(nm^2^‧ns), ∆*N* represents the increment of water molecules on the permeation side, ∆*t* is the total time of the simulation, and *S* is the sectional area of the nanopore.

The pressure within both the feed and permeation reservoirs is meticulously maintained using the constant force steered molecular dynamics (cfSMD) approach. This method, well-established in pressure control during non-equilibrium molecular dynamics simulations, involves applying a constant force to the fluid through a rigid plate that simulates a piston mechanism. The piston is constrained to move along the *Z* direction, effectively replicating the pressure application characteristic of a physical piston [31,32]. The force applied by the piston on each atom within the simulated region is maintained at a constant value, thereby ensuring accurate and stable pressure control across the reservoirs:

(3) $$F=P\cdot S_{pos}/N_{pos}$$

where *F* is the force applied to each piston atom, *P* is the applied pressure, *S_pos_* is the piston area, and *N_pos_* is the number of atoms composing the piston.

**Figure 14:**
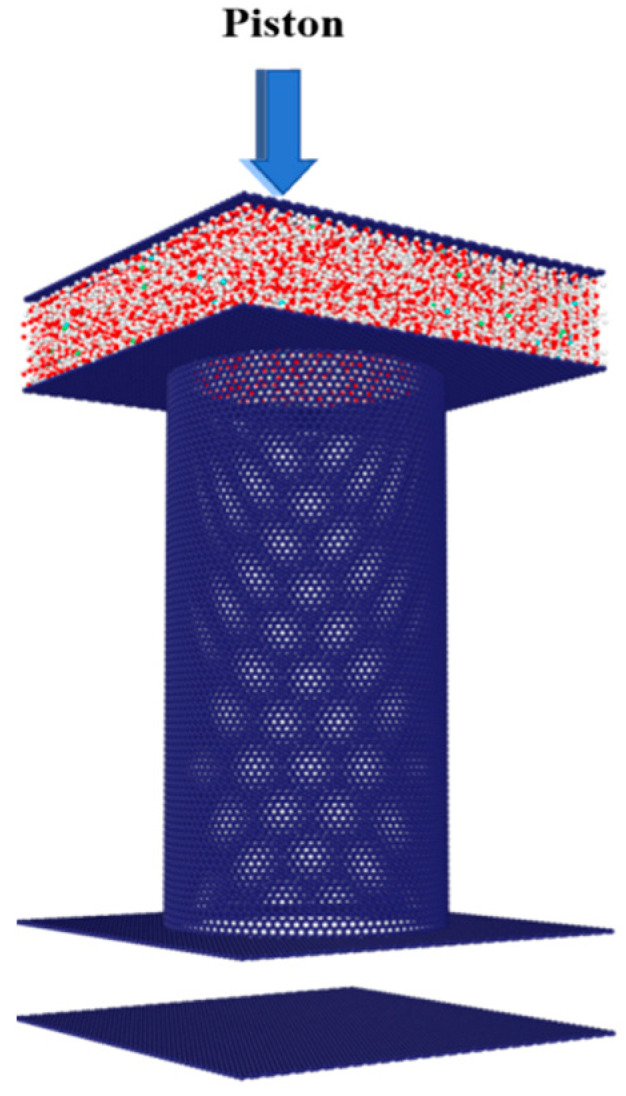
The physical model for calculating the LEP using the piston control method.

The hydrophobicity of membrane pores plays a crucial role in preventing the entry of liquid water. If the capillary pressure of the bent meniscus of liquid water formed at the entrance of the pore is lower than the pressure of the liquid water on the feed side, it will result in the ingress of liquid water into the pore, leading to pore wetting. To determine the minimum pressure required for a liquid to enter the pore, referred to as the liquid entry pressure (LEP), this study conducted molecular dynamics simulations to observe the wetting process of the pores under applied pressure, as depicted in Figure 14. The model shares geometric and thermal consistency with a DCMD model, except for the removal of the cold reservoir and a change in the pressure control method. Instead of maintaining a pressure of 1 atm by using a piston to drive the liquid through the system, the liquid is continually pressurized by the piston moving towards the membrane pores, thereby facilitating pore wetting. In non-equilibrium molecular dynamics simulations, a certain period is necessary for the system to stabilize and accurately record the pressure. Hence, the continuous piston movement process is divided into segments. Initially, the piston is displaced by 0.5 Å within 0.01 ns, after which it remains stationary while the system relaxes for 0.2 ns. During this 0.2 ns interval, the pressure exerted by the liquid on the piston is statistically sampled and averaged as the liquid pressure for that specific piston displacement. This process is then iterated, with the piston being displaced by 0.5 Å every 0.01 ns, followed by a 0.2 ns relaxation period until the membrane pores are completely wetted by the water.

**Figure 15:**
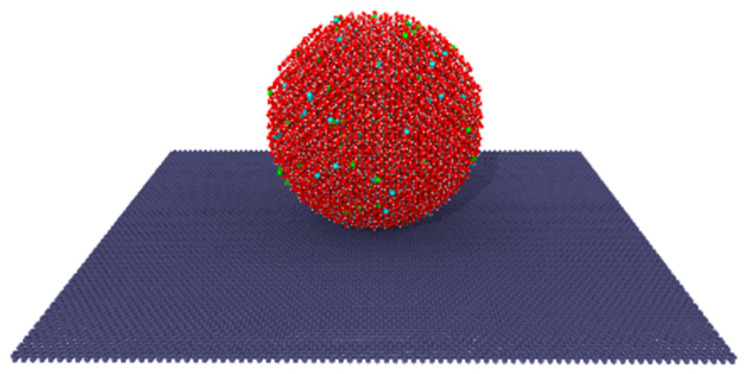
Contact angle testing model.

The contact angle testing model, as depicted in Figure 15, is constructed to visually illustrate the influence of salts on the wetting properties of the liquid on the membrane pores. The model consists of a single-layer graphene plate with dimensions of 200 Å × 200 Å and a solution droplet with a radius of 40 Å placed on the plate. Initially, water molecules are arranged according to a BCC 3.92 Å crystal structure, with salt ions randomly distributed within. Different solutions were equilibrated within the NVT ensemble at 300 K using a time step of 1 fs for 500 ps to obtain a stable structure with a stable contact angle. A homemade program was utilized to process the images of the stable contact angle, thus obtaining the contact angle of the liquid droplet. This approach provides a systematic and quantitative method for understanding how the salts influence the wetting properties of the liquid on the membrane pores.

## 4. Concluding Remarks

This study utilized non-equilibrium molecular dynamics to study the membrane distillation process and wetting processes in nanopores and analyzed the effects of NaCl concentration and different types of salts on membrane distillation performance. The main findings are as follows.

With the increase in NaCl concentration, the contact angle between the salt solution and the hydrophobic nanoporous membrane slightly decreases, leading to a reduced liquid entering pressure. Additionally, different types of salts, including CaCl_2_, KCl, LiCl, and NaCl, have minimal impact on the membrane wettability and liquid entering pressure.

The formation of hydrated ions is a critical factor affecting membrane flux. As the concentration of the salt solution increases, the hydrated structure of salt ions becomes more stable, leading to a reduction in membrane flux. Moreover, the first hydration shell radius of Ca^2+^ and Li^+^ is the smallest, and the peak of the *g*(*r*) between water molecules and cations is the highest, resulting in the lowest membrane flux for these salt solutions.

The concentration and type of salt solution are significant factors affecting the diffusion ability of water molecules in the feed reservoir. Higher salt concentrations weaken the diffusivity of water molecules, making evaporation more challenging. Additionally, water molecules in CaCl_2_ and LiCl solutions have weaker diffusion abilities compared to those in KCl and NaCl solutions.

## Figures and Tables

**Table 1:** Parameters of pair interaction.

	*ε*/(kcal‧mol^−1^)	σ/Å	Charge/e
O (H_2_O)	0.1553	3.166	−0.8476
H (H_2_O)	-	-	0.4238
C(Piston)-O (H_2_O)	0.15	3.358	-
C (membrane pores and surfaces)-O (H_2_O)	0.0576	3.358	-
Na^+^	0.1247	2.876	+1.0
Cl^−^	0.1247	3.785	−1.0
Li^+^	0.16494	1.505	+1.0
K^+^	0.1	3.331	+1.0
Ca^2+^	0.450	2.361	+2.0

**Table 2:** Composition of different feeds.

Liquid Feed	Number of H_2_O	Number of Salt Molecules	Molarity/(mol/L)
H_2_O	12,600	0	0
5% NaCl	12,600	204	0.85
10% NaCl	12,600	431	1.71
20% NaCl	12,600	970	3.42
LiCl	12,600	204	0.87
	12,600	431	1.76
	12,600	970	3.62
KCl	12,600	204	0.84
	12,600	431	1.66
	12,600	970	3.24
CaCl_2_	12,600	204	0.82
	12,600	431	1.57
	12,600	970	2.90

## Data Availability

The raw data supporting the conclusions of this article will be made available by the authors on request.
